# Reaching Older People With a Digital Fall Prevention Intervention in a Swedish Municipality Context—an Observational Study

**DOI:** 10.3389/fpubh.2022.857652

**Published:** 2022-04-25

**Authors:** Saranda Bajraktari, Magnus Zingmark, Beatrice Pettersson, Erik Rosendahl, Lillemor Lundin-Olsson, Marlene Sandlund

**Affiliations:** ^1^Department of Community Medicine and Rehabilitation, Physiotherapy, Umeå University, Umeå, Sweden; ^2^Municipality of Östersund, Health and Social Care Administration, Östersund, Sweden; ^3^Department of Epidemiology and Global Health, Umeå University, Umeå, Sweden

**Keywords:** accidental falls, aged, balance and strength exercise, digital health, mobile health, RE-AIM framework, reach

## Abstract

**Background:**

There is robust evidence that falls in old age can be prevented by exercise programs that include balance training, functional exercises, and strength training. For the interventions to have a population health impact, outreach to the population of focus with suitable interventions is needed. While digital interventions are promising there is limited knowledge on the characteristics of who is reached. The aim of this study was to describe the recruitment process, estimate reach rate at the population level and to describe participants characteristics and representativeness in a digital fall prevention intervention study.

**Methods:**

In a municipality-based observational study, reach of a digital fall prevention intervention was evaluated. The intervention included a digital exercise programme (Safe Step) and optional supportive strategies, complemented with a range of recruitment strategies to optimize reach. Recruitment during a period of 6 months was open to people 70 years or older who had experienced a fall or a decline in balance the past year. Reach was based on data from the baseline questionnaire including health and demographic characteristics of participants. Representativeness was estimated by comparing participants to a sample of older people from the Swedish National Public Health Survey.

**Results:**

The recruitment rate was 4.7% (*n* = 173) in relation to the estimated population of focus (*n* = 3,706). Most participants signed up within the first month of the intervention (*n* = 131). The intervention attracted primarily women, older people with high education, individuals who used the internet or digital applications almost every day and those perceiving their balance as fair or poor. Safe step participants lived more commonly alone and had higher education and better walking ability in comparison to the Swedish National Public Health Survey.

**Conclusions:**

With a range of recruitment strategies most participants were recruited to a digital fall intervention during the first month. The intervention attracted primarily highly educated women who frequently used the internet or smart technologies. In addition to digital fall prevention interventions, a higher diversity of intervention types (digital and non-digital) is more likely to reach a larger group of older people with different needs.

**Clinical Trial Registration:**

ClinicalTrials.gov, NCT04161625 (Retrospectively registered), https://clinicaltrials.gov/ct2/show/NCT04161625.

## Introduction

The population of older people in Sweden is increasing rapidly. Now more than ever, implementing health promotion and prevention interventions to support older people in maintaining independence and good quality of life is vital (1). Although many older people can expect more years of good health in later life compared to previous generations, the risk of developing disease or disability increases with higher age (2). Falls constitute a risk for injury and subsequent disability since fall rates increase in higher ages. At least one-third of community dwelling people aged 65 years and older fall every year; half of them fall more than once (3). In Sweden, fall-related injuries cause the highest number of deaths, hospitalizations, and visits to emergency services among older people (4). In addition to physical injuries, falls often lead to fear of falling and decreased balance confidence that negatively affect quality of life, e.g., through loss of functional independence, or avoidance of activities and participation in social events (5). Additionally, falls in older people are costly due to high costs for healthcare services, specialized care, and long-term social care needs such as home help or special housing (6).

The evidence-based fall prevention intervention strategies are robust, with many positive results identified by systematic reviews (7, 8). Falls are multifactorial in nature, with multiple interacting risk factors such as adverse drug effects, visual limitations, gait disturbances, nutrition, and impaired balance (9). Interventions consisting only of exercise that targets functioning, balance and strength are effective at reducing fall rates, fall risk and probably fall-related injuries among community dwelling older people (7).

Reaching the population of focus is crucial if these interventions are to be effective and have an impact on population health (10). Reach is a measure of participation and one of the five domains of the RE-AIM framework. The basis of RE-AIM is that the ultimate impact of an intervention is due to its combined effects on five evaluative domains: reach, efficacy, adoption, implementation, and maintenance (10). This study will focus on assessing the reach domain. Reach is defined as the absolute number, proportion, and representativeness of individuals who are willing to participate in a given initiative (10, 11). Reach can be assessed in various ways, e.g., before and/or post-intervention, and can consist of demographic characteristics of the sample, demographic characteristics of the population of focus, comparison of the sample to the population of focus (representatives), and description of recruitment strategies (11). Reaching older people with health promotion and preventive interventions is challenging (12). As observed in previous trials, about two-thirds of older people decline to participate in health promotion interventions for various reasons, such as lack of time/interest or poor health (13, 14). Such results have been also shown in exercise interventions. In a systematic review Nyman et al. (15) evaluated participation and adherence to single component fall prevention programs of different formats and found out that the acceptance rate to participate in exercise interventions was lower (64.2 % of people invited accepted the invitation) compared to other fall prevention interventions, e.g., interventions utilizing medication or home adoption interventions (acceptance rates of participation of 81.7 and 71.4% respectively) (15).

Traditional exercise interventions are commonly supervised by a trained professional and delivered either in a group (16) or an individual format (17). Considering limited resources, reach of many older people by traditional professional-led exercise intervention formats can be challenging (18). Therefore, digital formats might be a good complementary alternative to increase reach. With the support of digital technology, it may be possible to provide efficacious interventions to older people at lower costs, regardless of where they live. Furthermore, digital technology is a promising mode for tailored personalized exercise with the ability to support independent exercise, and the possibility of maintaining exercise over a longer period (19).

Delivering fall prevention exercise interventions through digital solutions is a rather new and emerging field. Several randomized controlled trials are ongoing and some have already reported promising results (20, 21). For example, Delbaere et al. (20) showed that a digitally driven and self-managed fall prevention exercise programme can result in a significant reduction in fall rates. That intervention had good adherence that ranged from 30–40% over 2 years. The Safe Step exercise application is currently under evaluation in a nationwide Swedish randomized controlled trial (22). Safe Step is a fully self-managed digital exercise programme developed in co-creation with older people who were 70 years of age or older (23). A four-month feasibility study showed that participants who exercised with the Safe Step were more satisfied with the support than participants exercising with a self-managed booklet format (24). One year after the start of the intervention, participants who exercised with the digital programme were more likely to continue to exercise regularly (67%) than participants in the booklet group (35%) (25). Nonetheless, in addition to the RCT, evaluation of reach for the Safe Step intervention in routine practice is necessary to better understand the potential to reach out with a digital intervention if implemented. Pragmatic study designs such as observational studies represent important study designs to better inform public health practice about this (26).

The aim of this study was to evaluate reach of the Safe Step digital fall prevention exercise intervention in people 70 years and older and living in their own homes (community-dwelling) in a Swedish municipality. Reach was assessed by means of (a) describing the recruitment process and evaluating the recruitment rate, (b) describing the characteristics of the Safe Step sample, and (c) determining the representativeness of the Safe Step sample compared to another sample of older people extracted from the Swedish National Public Health Survey (27).

## Methods

This study is based on data from the Safe Step observational study conducted in Östersund, a medium-sized municipality in Sweden (28). The municipality covers an area of 2,502 km^2^ and includes rural areas and one city. At the time of the study, the municipality had about 64,000 citizens with 21% being at least 65 years of age. The Safe Step study consisted of a one-year self-managed digital exercise programme, monthly educational videos on fall prevention, and optional supportive strategies such as introductory drop-in meetings, group exercise sessions, and technical support. More information on the intervention components is presented below. The study received ethical approval from the Swedish Ethical Review Authority (Dnr 2019/03763). The protocol is registered in ClinicalTrials.gov (NCT04161625). The study protocol is not published elsewhere. This study was guided by the STROBE checklist (Strengthening the Reporting of Observational studies in Epidemiology) (Supplementary Material 1) (29).

Inclusion criteria for this study included people 70 years or older living in the municipality of Östersund, history of a fall or experienced a perceived decline in postural balance during the previous year, access to a smartphone or tablet that was used regularly, active personal email address, ability to understand verbal and written instructions in Swedish, ability to rise from a standard height chair without assistance, and independent in indoor walking without a walking aid. An age limit of 70 and over is proposed as the target age group of fall prevention strategies due to the increased risk of falling with increased age (30). Exclusion criteria included progressive disease likely to lead to a decline in strength or balance over the next year, perceived memory dysfunction that affected everyday life activities, and participation in more than 3 h of strenuous physical exercise (e.g., dance, gymnastics, gym exercises, running or skiing) a week.

### Intervention Components and Strategies to Optimize Reach

All study participants were provided with free access to the Safe Step digital exercise programme and voluntary supportive strategies to use when needed.

#### Safe Step Digital Exercise Programme

Safe Step is a fully self-managed programme provided in a mobile application format. The foremost components are functional, balance and strength exercises that are categorized into 10 different exercise categories (four categories targeting lower-limb muscle strength, three categories targeting balance and three categories for gait and step exercises). Within each category, exercises are shown in several short videos that are ordered based on the difficulty level. Participants were instructed to create their individual programs by choosing one exercise from each category and that chosen exercises were to be challenging but not too hard. Participants were also asked to practice at least 30 minutes, 3 days a week and to progress to more advanced exercises when appropriate. Instructions for this were given in the videos and to be based on their own evaluations. The application includes “tools” to support behavior change in the form of an integrated calendar for planning and monitoring exercise activity (24). An integrated virtual physiotherapist provides reminders and feedback after each exercise session. More details on the Safe Step programme are published elsewhere (22). A more detailed description of the intervention can also be found in the Template for intervention Description and Replication (TIDieR) guide (Supplementary Material 2).

#### Optional Supportive Strategies

Support for exercising with Safe Step were developed in co-creation with a group of older people, a local non-profit organization (Friskis&Svettis) that offers gym and exercise classes, and the municipality of Östersund. The strategies included how to manage the technology, and a range of in-person supportive strategies (introductory drop-in meetings, group exercise sessions and technical support). While several supportive strategies were scheduled from the start of the intervention, more opportunities were planned to meet an eventual increase in requests. Nevertheless, interest for supportive strategies was low therefore no additional meetings were added.

##### Introductory Drop-in Meetings

The purpose with these meetings was to introduce the intervention to interested participants and to make it possible for registered participants to ask questions. The meetings took place at different senior centers in the municipality. Initially, eight introductory meetings were scheduled (1 h per meeting).

##### Group Exercise Sessions

During the first 7 months of the study, participants had the opportunity to attend three different group sessions to learn how to use the Safe Step programme. Group sessions were initially offered three times per week during the first 7 months of the study. Two authors (MS and LO) who are experienced physiotherapists, developed a detailed plan for the group exercise sessions and provided an introductory presentation concerning falls and falls prevention and an introduction to the Safe Step programme for Friskis and Svettis instructors, who later managed the group sessions. Group exercise sessions were organized in three stages. Stage one was a general introduction to the programme, stage two focused on the choice of exercises, and stage three focused on progression guidance.

##### Technical Support

Technical support was offered at different locations such as the city library and various senior meeting centers in the municipality of Östersund. Initially, 9 one-hour meetings were scheduled. Interested participants could drop in on scheduled dates and ask questions about the application and project and to get help with the technology (e.g., downloading the app, general app use). Drop-ins at the city library were arranged in collaboration with IT-Guide, an organization comprised of adolescents with knowledge in information and communication technology (ICT). IT-Guide offered drop-in opportunities for older people who needed help with technology (e.g., creating a Facebook account). We collaborated with IT Guide to provide technical support with the Safe step application. Drop-ins at senior meeting centres were hosted by one of the authors (SB) in collaboration with the municipality-based digital support department.

#### Recruitment

**Figure 1:**
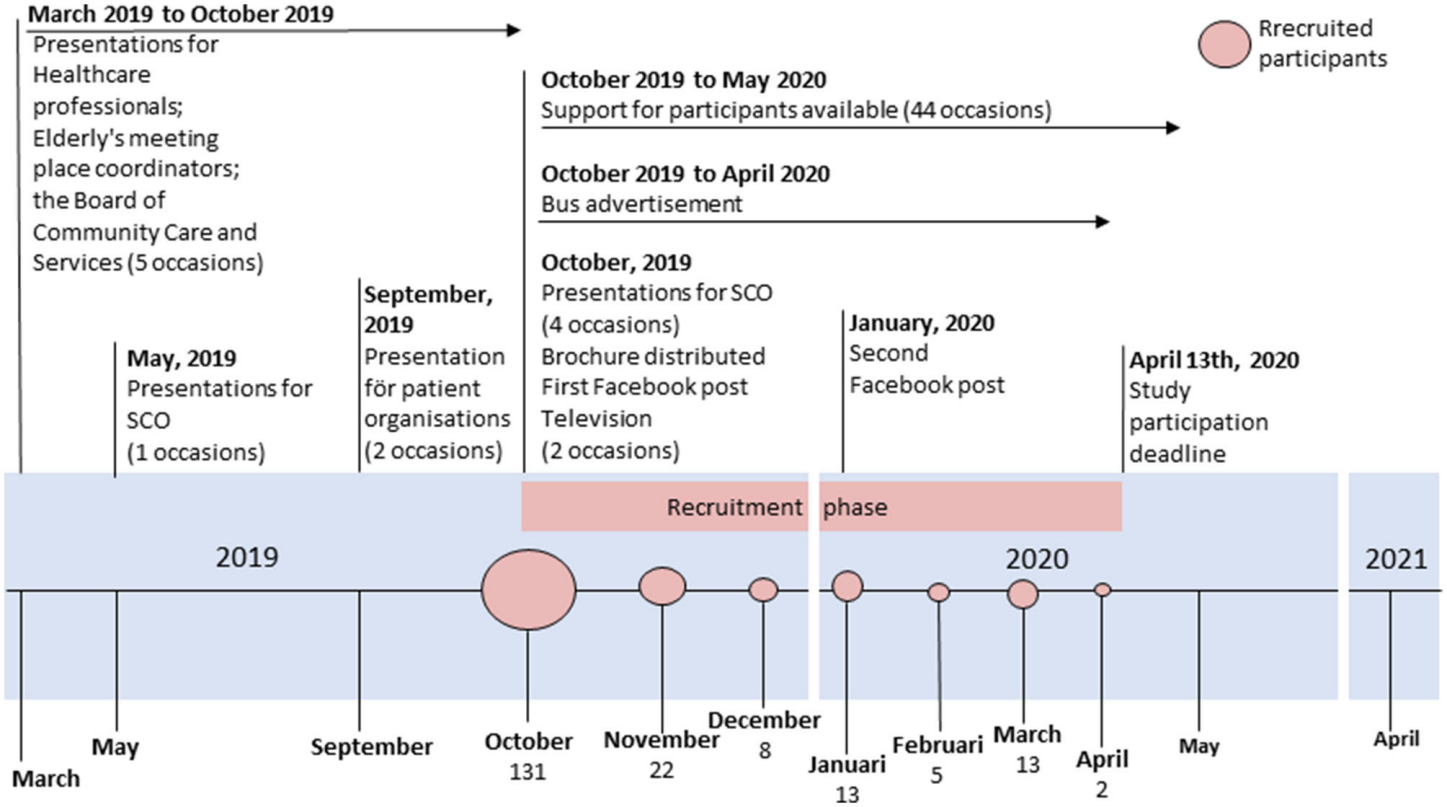
Timeline of recruitment strategies and total number of participants recruited. SCO = senior citizen organisation.

Participants were recruited over 6 months beginning on October 14, 2019. The recruitment goal was to reach as many as possible from the population of focus. Based on literature on recruitment strategies several options of strategies were presented, considered, and discussed with a reference group of older people (31, 32). The reference group consisted of active members of different senior citizen organizations who volunteered to participate after sending a participation inquiry by email. A range of 12 different digital or non-digital recruitment strategies were utilized. The recruitment strategies focused on informing about the importance of fall prevention and the opportunity to participate in the upcoming study. All recruitment strategies were utilized at approximately the same time. See Figure 1.

##### Digital Recruitment Strategies

Digital recruitment strategies included the municipality's official Facebook website, the homepage of the Municipality of Östersund, and TV screens commonly visible in waiting rooms at primary care centers. Facebook posts were published twice during the recruitment period: the first 2 weeks before recruitment began and the second one a month after recruitment started.

##### Non-digital Recruitment Strategies

Non-digital recruitment strategies included advertisements on buses, a brochure addressed to the population of focus and delivered by post, and several oral presentations. Two small advertisements with information about the study were placed on each local bus in the largest city of the municipality. The bus advertisements could be seen from outside and inside the bus. Close-to-final versions of the advertisements were presented to a group of older people. The three advertisements presented included different headings (translated here from Swedish) such as “*Afraid of falling?”*, “*Exercise to feel safer*” and “*Is your balance not like it used to be?”*. The consultation resulted in the decision to use all three advertisements since different persons could relate to the different tone of the message in the advertisements. Bus advertisements stayed up throughout the recruitment period.

The brochure was sent to each household that included at least one community-dwelling person aged 70 years or older, in all (*n* = 9,880 persons). The municipality provided a register (excel file) with addresses for the population of focus. The brochure included information about the Safe Step intervention and how to sign up for the study. In addition, as a separate topic from the intervention but related to fall prevention, other educational information on fall prevention was provided (i.e., information on falls risk factors such as medication use and nutrition; information on different services that the municipality provided).

Presentations that included information about the study and the intervention were held for groups such as senior citizen organizations, healthcare professionals working in the municipality or primary care, patient organizations, meeting places for older people organized by the municipality, and the Municipality Board of Community Care and Services (Figure 1). Many presentations were held approximately one month before the start of recruitment.

#### Enrolment and Participants

Regardless of the recruitment strategy, all participants were directed to the project's website for enrolment. All information about the study, procedures, consent, eligibility criteria, dates and locations for the different supportive strategies were available on the website. The website was built in collaboration with the municipality's department of media and communication and in co-creation with the reference group. Before registering for the study, interested participants assessed themselves whether they met the inclusion criteria. Those who judged themselves eligible and agreed to participate were enrolled by providing their email addresses.

#### Data Collection

Participants answered five web-based questionnaires during the 12-month intervention period (at baseline, 3, 6, 9, and 12 months follow up). Each questionnaire was sent to the participant through their personal email. Additionally, participants were asked to report any falls through a digital questionnaire sent monthly by email. In the same email, participants received a link to a short video lecture on a topic related to fall prevention, e.g., home safety, the importance of diet, medication, and balance. All questionnaires were based on self-reports and self-tests. For this study, only data from the baseline questionnaire is included.

The baseline questionnaire included questions on sex, age, weight, height, education, use of the internet, living conditions (residency, household), falls the past year, quality of life, medication use, diagnoses, perceived memory dysfunction, gait speed, level of physical activity, physical performance, self-reported balance and strength, self-reported improvement in balance and strength in comparison to the previous year, fear of falling, current physical activity behavior, and information about recruitment pathways. Quality of life was assessed by the 5-dimension, European Quality of Life questionnaire (EQ-5D-5L) (33). Level of physical activity was measured by two questions with six predetermined alternatives (minutes/week of physical daily activities and minutes/week of strenuous physical activity). This measure is based on the standard measure of physical activity level used in the Swedish National Public Health Survey, therefore practical for the Swedish context and comparison of the study sample with the general population of older people. Physical performance was measured by a modified self-administered version of the 30 s chair stand test (30CST) (34). Participants were instructed through the web-based questionnaires in performing the self-administered test. The instructions have been developed by the research group and were advised with a group of seniors before finalizing the instructions (22). Fear of falling was measured by the Swedish version of the Falls Efficacy Scale-International (FES-I) (35). Current physical activity behavior was evaluated by asking participants to choose one of the five claims that best matched their behavior. The claim categories are based on the transtheoretical model and summarized as follows: “not engaging in regular exercise and no intention to start (in the near future)” (precontemplation), “strongly considering making changes” (contemplation), “making small changes” (preparation), “engaging in exercise for <6 months” (action), or “exercising regularly for at least 6 months” (maintenance phase) (36).

Recruitment pathways were identified by asking participants to answer a question on how they learned about the study. Thirteen options were provided, including one free-text option. To identify the most influential pathway, participants could choose only one option. Notes on attendance numbers for the recruitment and supportive strategies were kept by the person in charge of performing the specific presentations or hosting the supportive strategies (SB, MZ or representative from Friskis&Svettis).

To compare the Safe Step sample with a representative sample of people aged 70+ living in Östersund, data on gender, household situation, general health, and functional performance (ability to take walks of about 5 min) were extracted from the National Public Health Survey issued by The Public Health Agency of Sweden (27). The National Public Health Survey is conducted biannually in Sweden and once in 4 years the survey is sent to a larger number of people living in certain parts of Sweden, for example the county of Jämtland Härjedalen, including the municipality of Östersund.

#### Data Analyses

The total number of persons in the population of focus was estimated from available statistics using the study inclusion and exclusion criteria. Those living in special housing (*n* = 508) (37) were subtracted from the total number of people 70 years or older living in the municipality in 2018 (*n* = 9,800) (38). Based on data available for internet use on mobile phones in older people, we estimated that 77% of those living in their own homes (*n* = 9,292) used internet on mobile devices, i.e., mobile phones and tablets. (39). Based on statistics on physical activity levels in older people 70+, we estimated that 51.8% did not exercise more than 150 min/week, e.g., an estimate related to the exclusion criteria (Exercising more than 3h/week) (27). Using the above statistics, the population of focus included 3706 individuals. The recruitment rate was calculated by dividing the total number of Safe Step participants (numerator) by the total number of people in the estimated population of focus (denominator) (10).

Descriptive statistics were performed to describe participant characteristics, using means and standard deviations for continuous data and proportions and frequencies for categorical data. Descriptive statistics were also used to summarize results of recruitment pathways. Men and women were compared using the Mann-Whitney *U*-test for comparison of continuous measures and Pearson Chi-square test for comparison of categorical measures. In the analysis, three illogical extreme outliers related to the variables of weight and number of falls were excluded.

Pearson Chi square test was used to compare characteristics of the Safe Step sample and another cohort of people aged 70+ living in Östersund who answered the National Public Health Survey. Analyses were performed with SPSS Statistics 25 and the significance level was set at α ≤ 0.05.

## Results

### Recruitment of Participants

Enrolment of participants was finalized on 13 April 2020. In total, 194 participants registered for the intervention, 173 answered the baseline questionnaire, and those 173 were included in the study (Figure 1). Recruitment over time showed a clear decreasing trend (Figure 1), with most participants signing up within the first month of the intervention (*n* = 131). The recruitment efforts yielded a recruitment rate of 4.7 % in relation to the estimated population of focus (*n* = 173/3706).

The recruitment strategies were implemented as planned. Interest from media led to two short reports on the local television station. For recruitment purposes, the study was presented on 12 different occasions (Figure 1). A total of 382 persons attended the presentations for senior organizations or patient organizations, and 82 individuals attended presentations targeted at personnel working with older people. The most frequently reported source of recruitment was the brochure (47%, *n* = 81) followed by older adults' meeting place coordinators (13%, *n* = 22). Fewer reported other strategies such as social media (11%, *n* = 19), newspaper, television, and radio (8%), information from family or acquaintance (5%), municipality staff (4%), senior meeting centers, Friskis&Svettis, and patient associations (2% each), municipality homepage (1%), and a few did not remember or did not answer the question (4%).

### Safe-Step Sample Characteristics

Self-reported participant characteristics are presented in Table 1. The majority of participants were women, had higher education and used the internet or smart technology applications several times per day. The majority of participants reported living in the city, cohabited with one or more persons and reported having fair or poor balance. Fewer than half experienced a deterioration in balance the past year and slightly less than half of all participants had sustained a fall in the last year. About 32% (*n* = 56) had neither experienced a fall nor a deterioration in balance the past year (responding “better” the question on “Change in perceived balance the past year” and reporting no falls the past year). The few 3.5% (*n* = 6) had reported no falls and no concerns at all with balance in comparison to the past year (responding “better” or “approximately the same” the question on “Change in perceived balance the past year” and reporting no falls the past year). Slightly less than half of participants reported not exercising regularly (participants not in the maintenance or action phase of the transtheoretical model of behavior change).

**Table 1:** Participant's characteristics.

**Variable**	**Total**	**Women**	**Men**	***p*-value**
	***N =* 173**	***n =* 121**	***n =* 51**
	**100%**	**70.3%**	**29.4%**
**Age**, mean ± SD (min–max)	76.1 ± 4.7 (70.0–93)	75.79 ± 4.54 (70–93)	76.84 ± 4.96 (70–90)	0.22
**BMI**, mean ± SD	26.28 ± 4.16	26.54 ± 4.47	25.69 ± 3.25	0.36
**Education**, ***n*** **(%)**				0.37
1–9 years	31 (17.9)	25 (20.7)	6 (11.8)	
10–12 years	42 (24.3)	29 (24.0)	13 (25.5)	
12+ years	99 (57.2)	67 (55.4)	32 (62.7)	
**Use of internet or applications on smart technology**, ***n*** **(%)**				0.04
Multiple times per day	91 (52.9)	56 (46.3)	35 (68.6)	
Almost every day or at least once per week	71 (41.3)	56 (46.3)	15 (29.4)	
At least once per month but not every week, or more seldom	4 (2.3)	4 (3.3)	0	
Not at all for the last 3 months or never	6 (3.5)	5 (4.1)	1 (2.0)	
**Residency**, ***n*** **(%)**				0.28
City	143 (83.1)	103 (85.1)	40 (78.4)	
**Household**, ***n*** **(%)**
Live alone	64 (37.2)	54 (44.6)	10 (19.6)	**0.002**
**Falls the past year**				
Number of individuals reporting fall, *n* (%)	77 (44.5)	52 (42.9)	24 (47.1)	0.62
Falls per person-year, (mean ± SD)	1.03 ± 2.731	1.11 ± 3.178	0.84 ± 1.173	0.32
Number of individuals with 1 or more falls requiring medical treatment, *n* (%)	25 (14.5)	18 (14.9)	6 (11.8)	0.59
Number of individuals with 1 or more indoor falls, *n* (%)	35 (20.2)	25 (20.7)	9 (17.6)	0.65
**Fear of falling, median [IQR]**	22 (7)	23 (8)	21 (7)	**0.029**
**Self-rated overall health**, ***n*** **(%)**				0.98
Very good or good	83 (48.0)	58 (47.9)	24 (47.1)	
Fair	84 (48.6)	59 (48.8)	25 (49.0)	
Poor or very poor	6 (3.5)	4 (3.3)	2 (3.9)	
**Health related quality of life**				
EQ-5D-5L VAS^2^, mean ± SD	69.60 ± 15.50	69.81 ± 15.35	69.29 ± 16.12	0.90
EQ-5D-5L index^3^, median [IQR]	0.859 [0.189]	0.8 [0.197]	0.859 [0.147]	0.54
**Prescription medications/day**, *n* (%)
4+	60 (34.7)	37 (30.6)	22 (43.1)	0.11
Medical conditions^*^, ***n*** **(%)**				
Discomfort, muscles and joints	72 (41.6)	60 (49.6)	12 (23.5)	**0.002**
Rheumatism	65 (37.6)	49 (40.5)	15 (29.4)	0.20
Eye disease	34 (19.7)	28 (23.1)	5 (9.8)	**0.042**
Cardiovascular disease	34 (19.7)	19 (15.7)	15 (29.4)	**0.039**
Incontinence	28 (16.2)	23 (19.0)	4 (7.8)	0.06
Dizziness	27 (15.6)	21 (17.3)	6 (11.8)	0.35
Diabetes	22 (12.7)	11 (9.1)	11 (21.6)	**0.025**
Lung disease	20 (11.6)	15 (12.4)	4 (7.8)	0.38
Osteoporosis	20 (11.6)	18 (14.9)	1 (2.0)	**0.014**
**Experience of memory dysfunction**, ***n*** **(%)**				
No	92 (53.2)	71 (58.7)	20 (39.2)	**0.020**
**Gait speed compared to others in the same age**, ***n*** **(%)**				0.80
Faster	47 (27.2)	32 (26.4)	14 (27.5)	
As fast	59 (34.1)	40 (33.1)	19 (37.3)	
Slower	67 (38.7)	49 (35.3)	18 (35.3)	
**Physical performance, mean** **±SD**				**0.17**
30-second Chair-Stand Test	12.56 ± 4.83	13.31 ± 4.86	12.25 ± 4.83	
**Perceived balance**, ***n*** **(%)**				0.94
Very good or good	41 (23.7)	29 (24.0)	12 (23.5)	
Fair	89 (51.4)	61 (50.4)	27 (52.9)	
Poor or very poor	43 (24.9)	31 (25.6)	12 (23.5)	
**Change in perceived balance the past year**, ***n*** **(%)**				0.10
Better	10 (5.8)	8 (6.6)	1 (2.0)	
Approximately the same	85 (49.1)	64 (52.9)	21 (41.2)	
Worse	78 (45.1)	49 (40.5)	29 (56.9)	
**Perceived leg strength**, ***n*** **(%)**				0.38
Very good or good	59 (34.1)	41 (33.9)	17 (33.3)	
Fair	84 (48.6)	56 (46.3)	28 (54.9)	
Poor or very poor	30 (17.3)	24 (19.8)	6 (11.8)	
**Change in perceived leg strength the past year**, ***n*** **(%)**				0.98
Better	8 (4.6)	5 (4.1)	2 (3.9)	
Approximately the same	113 (65.3)	79 (65.3)	34 (66.7)	
Worse	52 (30.1)	37 (30.6)	15 (29.4)	
**Walking aids**, ***n*** **(%)**	22 (12.7)	16 (13.2)	6 (11.8)	0.79
**Physical activity**, ***n*** **(%)**				
>2 h/week physical daily activities	40 (23.1)	31 (25.6)	8 (15.7)	0.85
>2 h/week strenuous physical activity	19 (11.0)	13 (10.7)	5 (9.8)	0.15
**Transtheoretical model**, ***n*** **(%)**				0.91
Maintenance	81 (46.8)	57 (47.1)	23 (45.1)	
Action	14 (8.1)	9 (7.4)	5 (9.8)	
Preparation	23 (13.3)	15 (12.4)	8 (15.7)	
Contemplation	30 (17.3)	21 (17.4)	9 (17.6)	
Precontemplation	25 (14.5)	19 (15.7)	6 (11.8)	

*In bold, significant values ≤0.05*.

* *Medical conditions reported by ≥11% of participants. Conditions reported by <11% include e.g., arthritis, celiac disease, restless legs, sleeping problems. One missing observation per variable: Gender, Education, Use of Internet or applications on smart technology, Household. Two unjustifiable extreme values in relation to number of falls the past year have been excluded from the analysis*.

Sample characteristics were similar between men and women. However, there were differences regarding the pattern of internet use (*p* = 0.04) even though both groups were frequent internet users (*p* = 0.06). Furthermore, women lived alone more often (*p* = 0.002) and reported higher ratings of fear of falling (*p* = 0.029). Compared to men, women reported memory dysfunction less often (*p* = 0.02).

### Sample Representativeness

In comparison to the National Public Health Survey, the Safe Step participants were more likely to have a higher education, live alone, have a better functional performance and were more likely to be women. For details, refer to Table 2.

**Table 2:** Distribution of self-reported characteristics of the Safe Step sample and National Public Health Survey (NPHS).

**Variable**	**Safe step**	**NPHS**	**p-value^a^**
**Age, Mean (min–max)**	76.10 (70–93)	75.27 (70–84)	<0.05
**Gender**, ***n*** **(%) Women**	121 (70.3%)	4,458 (54.0%)	<0.001
**Education**, ***n*** (%)			<0.001
1–9 years	31 (18.0%)	1,818 (22.0%)	
10–12 years	42 (24.4%)	4,110 (49.8%)	
More than 12 years	99 (57.6%)	2,322 (28.1%)	
**Household situation**, ***n*** (%)			<0.001
Living alone	64 (37.2%)	2,942 (24.8%)	
**General health**, ***n*** (%)			<0.005
Very good or good	83 (48.0%)	4,457 (54.5%)	
Fair	84 (48.6%)	3,175 (38.8%)	
Poor or very poor	6 (3.5%)	546 (6.7%)	
**Walking ability**, ***n*** (%)			<0.001
Can walk about 5 min	156 (90.2%)	3,739 (77.3%)	

a *Chi square*.

*NPHS, National Public Health Survey*.

Men and women from the Safe Step study were compared separately with the National Public Health Survey sample. Both men and women in the Safe Step sample had a higher education (men: 67.7 vs. 22.7 %, *p* < 0.001; women, 55.4 vs. 32.8 %, *p* < 0.001) and were more likely to take walks of about 5 minutes (women: 87.6 vs. 77.1 %, *p* = 0.005 and men: 96.1 vs. 77.6 %, *p* < 0.001). Significantly more women in Safe Step were living alone compared to women from the National Public Health Survey (44.6 vs. 29.8 %, *p* < 0.001).

## Discussion

Those recruited for this study were not entirely a representative sample of the population of focus: they were highly educated, used the internet or applications on smart technologies almost daily, and the majority were women. Most participants were recruited during the first month of recruitment and there was a 4.7 % recruitment rate in relation to the estimated population of focus.

Determining if a 4.7% recruitment rate is low or high is challenging. The number of actual participants is easily presented and can easily be compared with different studies (11). A challenge in comparing recruitment rates across studies is that different approaches have been used to describe the denominator (i.e., the population of focus). Most often, intervention studies do not evaluate reach the way we did but report possible equivalents to the denominators such as the total number of approached participants, the total number of approached and eligible participants, or the total number of approached populations who were eligible and signed up for the study (15, 40, 41). The number of approached individuals cannot be determined for the present study because of the recruitment strategies employed. The denominator was estimated from a broader perspective—the population of focus estimation was based upon the general population of people 70 years or older living in ordinary housing combined with statistics about internet use and physical activity levels in this population. Nevertheless, it was not always possible to extract statistics on older people of age 70 years and above, therefore some estimations such as internet use on mobile phones and persons living in special housing are based on older people of age 65 years and above. Thus, our approach to evaluating reach has limitations. Estimating recruitment rates in different ways will result in different, non-comparable rates. Harden et al. assessed reach of a home-based physical activity intervention for older people from two different perspectives. With the first perspective, the population of focus represented an estimation of the total number exposed to recruitment and resulted in a recruitment rate of 0.3% (41). With the second perspective, the population of focus consisted of those contacted and eligible and resulted in a reach rate of 80% (41). Reach can also be estimated on different levels and at different stages of the intervention period, and therefore it is of crucial importance to describe properly how reach was assessed. A recent systematic review included 21 studies addressing balance disorder in older people by means of Electronic Health (eHealth). The number of participants in these studies ranged from 16 to 196 (42). In comparison, the Safe Step intervention (*n* = 173) had a relatively high number of participants. Nevertheless, the previously mentioned review findings cannot be discussed in terms of reach on the population level since no such information is available.

Twelve different recruitment strategies were utilized for participant recruitment in this study. Previous research has called for the use of multiple and diverse recruitment strategies to increase the likelihood of reaching a diverse group of older people (31, 32). Older people acquire information in different ways, e.g., some prefer visual information, others prefer printed material, and some prefer both (31, 32, 43, 44). The heterogeneity in preferences was observed with all recruitment alternatives reported by at least one study participant as the way they learned about the study. Some strategies were more commonly reported than others, for example, the brochure was the most effective strategy. Traditional recruitment strategies such as direct mailing or personal contact with health professionals are effective in recruiting older people (42, 45, 46). All recruitment strategies could easily be implemented in ordinary practice. However, the development and distribution of a brochure as well as advertisement on buses comes with costs. An ongoing health economic evaluation will focus on the cost effectiveness of the Safe Step intervention. More specifically, we will explore if the resources required for recruitment strategies stand in reasonable relation to improve reach and subsequent impact on health effects in the population. For this study, all recruitment strategies were introduced concurrently, and participants could choose only one alternative on the baseline questionnaire (Figure 1). This restriction might have influenced participants to choose the most recent or easiest strategy to remember, and so the effect of the other strategies might be underestimated. Also, this reporting restriction limits the ability to account for the impact that a combination of recruitment strategies might have. Only by following the recruitment sequel can we suggest that several concurrent recruitment pathways might have increased exposure and awareness and thereby explain the larger number of participants (64 %) recruited during the first months of the six-month recruitment period.

The majority of Safe Step participants were highly educated and reported better walking ability than comparators in the National Health Survey sample. Consistent with the literature, participants with higher education are more likely to participate in research studies in general and those with health literacy proficiency are more likely to participate in digital interventions (47). Older people represent the fastest growing group of internet users in Sweden, with 87% of people in the ages of 65–74 years old and 73% aged of 75 years and older reported internet use in 2020 (48). Many access the internet by computer, but increasingly, older people are using smartphones for this purpose (49). However, this group lags behind all other groups and are at highest risk of digital exclusion (50). Those at greatest risk of digital exclusion are women, those with lower education, those who live in communities with lower socioeconomic status, and those who live alone (48). Inconsistent with the literature on groups at risk for digital exclusion (9, 51), the majority of Safe Step participants were women (70%), and they were more likely to live alone. Furthermore, Safe Step participants reported better walking ability in comparison to the National Health Survey sample. Those with functional ability are also more likely to participate in physical activity-based fall prevention interventions (52). To increase reach with fall prevention interventions, additional strategies are needed to attract older people with poorer health, those who have less digital access and fluency, and probably those with lower education (43, 53, 54).

Most participants in fall prevention interventions are women, and that was the case also in this study. One possible explanation is that both men and women perceive fall prevention interventions as more appropriate and beneficial for women (55). Women experience injurious falls more often than men (56) and therefore might be more motivated to participate in fall prevention interventions. Because the rate of fatal falls in men of all ages is significantly higher than for women (57), further investigation of how to reach men is important. An interesting approach could be to explore different marketing alternatives. As observed in this study, the reference group involved in the planning of the intervention (consisting of both men and women) preferred the use of different messages for recruitment advertising. Marketing falls prevention interventions while highlighting other benefits might increase the number of participating older men. Nevertheless, more research is needed to identify possible gender differences in preferences for recruitment strategies and fall prevention interventions (55).

One methodological implication worth discussing is the self-assessment of the inclusion criteria. This might explain the number of participants who would not meet all the inclusion criteria if assessment had been done by a professional. For example, 32% of participants had not experienced a fall or a deterioration in balance the past year and more than 50% of participants were already in the maintenance or action phase of the transtheoretical model (regularly physically active) (36). Still, self-assessment of inclusion criteria is a resource efficient way to include large numbers of participants in a short period of time and a good indicator who might participate if this was implemented in routine practice.

The Safe step intervention was designed as a self-administered intervention. Our findings indicate that the intervention can be administered without additional support and can be made freely available to older people in the community. While aspects of effectiveness and cost-effectiveness are being evaluated, in future studies, it is also important to evaluate (22) other components of the RE-AIM framework, for example, adaption which is crucial for the sustainability of the intervention when implemented in routine practice (10).

## Conclusions

With a range of recruitment strategies most participants were recruited to a digital fall intervention during the first month. The digital intervention attracted primarily highly educated women who frequently used the internet or smart technologies. This study did not find a single best solution to increase reach with a fall prevention intervention. A variety of digital and non-digital interventions complemented with a variety of recruitment strategies is likely needed to reach a larger and more diverse group of older people with different needs.

## Data Availability Statement

The datasets used for the current study are available on reasonable request.

## Ethics Statement

The intervention received ethical approval from the Swedish Ethical Review Authority (Dnr 2019/03763). All participants in the intervention gave ethics-approved informed consent prior to enrolling in the trial.

## Author Contributions

SB was involved in planning and conducting the study, drafting the manuscript with contributions from all authors, analyses, and interpretation of the results. MZ was involved in planning and conducting the study, interpretation of results, providing input, and editing the manuscript. BP was involved in the development of the first draft, analyses, and providing input. LL-O and ER were involved in planning the study, interpretation of results, and providing input. MS was involved in planning and conducting the study, analyses, interpretation of results, providing input, and editing the manuscript. Original study concept was developed by MZ and MS. All authors read and approved the final version of the manuscript.

## Funding

This work was financially supported by the Swedish Research Council for Health, Working Life and Welfare (FORTE, Dnr 2020-00589), and the Seniorhusen Foundation. Salary of the doctoral student (SB) is partially financed by Umeå University's Industrial Doctoral School for Research and Innovation (IDS). None of the financial contributors has had any influence in the design of the study nor the collection, analysis, or interpretation of the data. None has been involved in the manuscript writing process.

## Conflict of Interest

The authors declare that the research was conducted in the absence of any commercial or financial relationships that could be construed as a potential conflict of interest.

## Publisher's Note

All claims expressed in this article are solely those of the authors and do not necessarily represent those of their affiliated organizations, or those of the publisher, the editors and the reviewers. Any product that may be evaluated in this article, or claim that may be made by its manufacturer, is not guaranteed or endorsed by the publisher.

## Abbreviations

NHS: National Public Health Survey
RE-AIM: Reach, Effectiveness-Adoption, Implementation, Maintenance
KOLADA: Swedish Municipalities and County Councils database
BMI: Body Mass Index
eHealth: Electronic Health
SCO: senior citizen organization.
