# Supplementary material for: Reaching Older People With a Digital Fall Prevention Intervention in a Swedish Municipality Context—an Observational Study
Source: Front Public Health. 2022 Apr 25;10:857652. doi: 10.3389/fpubh.2022.857652 (PMC9082637; doi:10.3389/fpubh.2022.857652)
Supplement: Supplementary file 2 [file Data_Sheet_2.DOCX]

Supplementary Material

# Supplementary file 2. Template for Intervention Description and Replication guide (TIDieR)

| Item no./Name | Description |
| --- | --- |
| 1. Brief name | A self-managed digital exercise programme “Safe step” complemented with additional support. |
| 2. Why | This digital self-managed exercise programme has been developed in co-creation with older people with the premise of increasing access to effective fall prevention interventions for older people. Additional support was provided with the premise of increasing reach. |
| 3. What: Materials | The Safe Step intervention consists of a digital exercise programme and includes exercises inspired by the Otago Exercise Programme (OEP) and the Falls management Programme. Furthermore, voluntary additional support (help with technical aspects of the application and exercise introductions) has been provided to interested participants. All information on the intervention and registration procedures were available on the project’s website.  The Safe step exercise programme included:   1. A repository of exercises categorised into 10 groups with focus in balance, lower-limb strength, and gait/step (3 groups, 4 groups, and 3 groups). Each exercise is shown in a short video format including verbal instructions. 2. Each exercise group includes a description of the relevance of the exercise, safety aspects and how to adjust exercises in case of pain or other difficulties with performance. 3. Tips on how to perform exercise outdoors and how to incorporate exercise in everyday live, also shown in a video format. 4. Behaviour change support in the format of an exercise diary including a planning tool for weekly exercising, registration of performed exercises, statistics on performance and a virtual physiotherapist, providing reminders and motivational feedback adapted to personal preferences.   Voluntary support:   1. Drop-in meetings to introduce the intervention to interested participants. 2. Group exercise sessions to provide participants the opportunity to get an explanation of how to use exercises and tips on progression. 3. Technical support, provided in the format of drop-ins, where participants could drop in on scheduled dates and get help with the technology aspect of the programme (the mobile application) such as: how to download the application or how to navigate it. |
| 4. What: Procedures | Interested participants judged themselves if they would be eligible to participate in the intervention based on inclusion and exclusion criteria provided at the project website and could register for the study by providing their e-mail address at the website. Everyone who after registration answered the baseline questionnaire were included in the study and got access to the exercise application, which could be downloaded from application stores after providing a personal code. The personal code was created by the research group and was sent per e-mail to each participant.  Participants interested of Voluntary support could drop-in at different occasions. Several time slots were available. The schedule and venue for the different time slots was also available at the project’s website. |
| 5. Who provided | Participants used the Safe step exercise programme independently during the one-year intervention period.  Introductory drop-in meetings were provided by two of the authors.  Group exercise sessions were provided by a local wellness association (Friskis&Svettis).  Technical support was provided by different people including one of the study authors (background in public health), IT-Guide, an organisation consisting of adolescents with knowledge in information and communication technology (ICT) and a person working for the municipality-based digital support department. |
| 6. How | Included participants got full access to the exercise programme over the course of the intervention period (one year). With help of an initial introductory video shown the first-time participants activated the application, participants got guided on how to create their individual exercise programme by choosing one exercise from each group (10 groups).  Voluntary support was provided in person. Exercise sessions in a group format and drop-in meetings in person or group format, depending on the number of participants showing up at the scheduled time slots. |
| 7. Where | The intervention was evaluated in a municipality in the north of Sweden.  Participants could choose on their own where to perform the exercises. Nevertheless, each exercise video showed an older person exercising in their own home environment.  Exercise sessions were held primarily at the local wellness association location (Friskis&Svettis) but also at senior meeting centres run by the municipality where the intervention took place.  Drop-in meetings were held at the senior meeting centres run by the municipality where the intervention took place. |
| 8. When and how much | Participants were recommended to exercise at least 30 minutes 3 times per week (for one year) and to progress their programme according to their ability and with support in the digital application.  Voluntary support was optional. Initially from start of the intervention (October 2019) eight introductory meetings were scheduled. Group exercise sessions were arranged three times per week the first seven months of the intervention and technical support was provided at nine occasions. |
| 9. Tailoring | Participants were instructed to compose their own programme by choosing exercises that were challenging but manageable without risking losing balance. The chosen exercise could be changed at any time. |
| 10. Modifications | N/A |
| 11. How well: Actual | N/A |

Note: N/A (Not applicable)
